# Crystal structure of *N*,*N*-dimethyl-2-[(4-methyl­benz­yl)sulfon­yl]ethanamine

**DOI:** 10.1107/S2056989015010233

**Published:** 2015-06-06

**Authors:** Alan R. Kennedy, Abedawn I. Khalaf, Fraser J. Scott, Colin J. Suckling

**Affiliations:** a Westchem, Department of Pure & Applied Chemistry, University of Strathclyde, 295 Cathedral Street, Glasgow G1 1XL, Scotland

**Keywords:** crystal structures, sulfone, collagen-induced arthritis, non-classical hydrogen bonding

## Abstract

In the crystal, the title compound, C_12_H_19_NO_2_S, has a disordered structure with two equally populated conformations of the amine fragment. A pair of weak C—H⋯O inter­molecular inter­actions between the CH_2_ and SO_2_ groups gives a one-dimensional supra­molecular structure that propagates through translation along the *a*-axis direction.

## Abstract 2

The title compound has a disordered structure with two equally populated conformations of the amine fragment. A pair of weak C—H⋯O inter­molecular inter­actions between the CH_2_ and SO_2_ groups gives a one-dimensional supra­molecular structure running along the *a-*axis direction.

## Chemical context   

Parasitic helminths possess a number of evolutionary strategies that facilitate their co-existence with their host and, as such, up to one third of the global population may suffer from helminthetic infections (de Silva *et al.*, 2003 ▸). These parasites can secrete immunomodulatory mol­ecules that prevent the parasites’ clearance from the host without leaving the host vulnerable to opportunistic infections (Hewitson *et al.*, 2009 ▸). ES-62 is one such immunomodulatory mol­ecule, a protein, which was discovered in the secretions of the rodent filarial nematode *Acanthocheilonema* and demonstrated to induce an anti-inflammatory immunological phenotype (Harnett *et al.*, 1989 ▸). ES-62 has been studied for its potential to treat human diseases relating to inflammation, for example collagen-induced arthritis or rheumatoid arthritis, and many positive outcomes have been demonstrated. 

**Figure 1:**
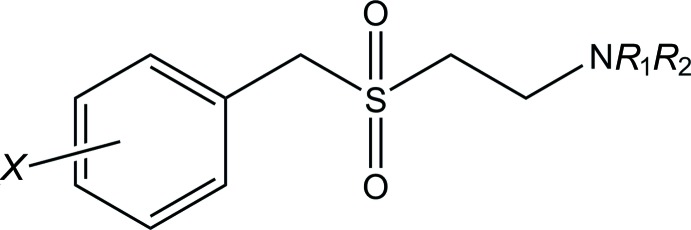
General structure of sulfone analogues. *R* represents alkyl chains and *X* represents halogen substituents.

A number of the significant anti-inflammatory activities of ES-62 are associated with post-translational glycosyl­ation and subsequent esterification by phospho­rylcholine. However, ES-62 is an immunogenic protein and is thus unsuitable as a drug itself (Harnett & Harnett, 2009 ▸). We have sought to capitalize on the immuno­modulatory effects of ES-62 whilst avoiding its inherent undrugability through synthesizing a library of drug-like small mol­ecules based upon phospho­rylcholine, the active moiety of ES-62. A series of sulfone analogues (Fig. 1 ▸) have proven to be of great significance in our investigations into collagen-induced arthritis. Despite the apparent simplicity of these mol­ecules, we are aware of no relevant crystallographic study. As such, and as the title compound is of particular inter­est to our ongoing work (Al-Riyami *et al.*, 2013 ▸), we report herein on the solid-state structure of the title compound.
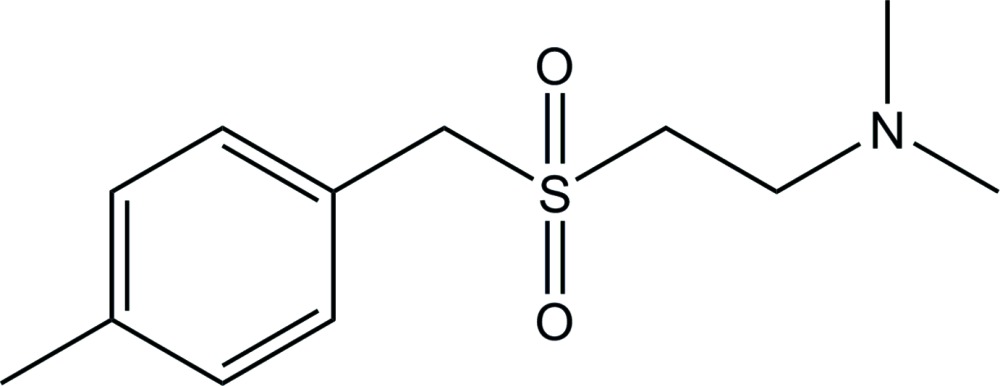


## Structural commentary   

**Figure 2:**
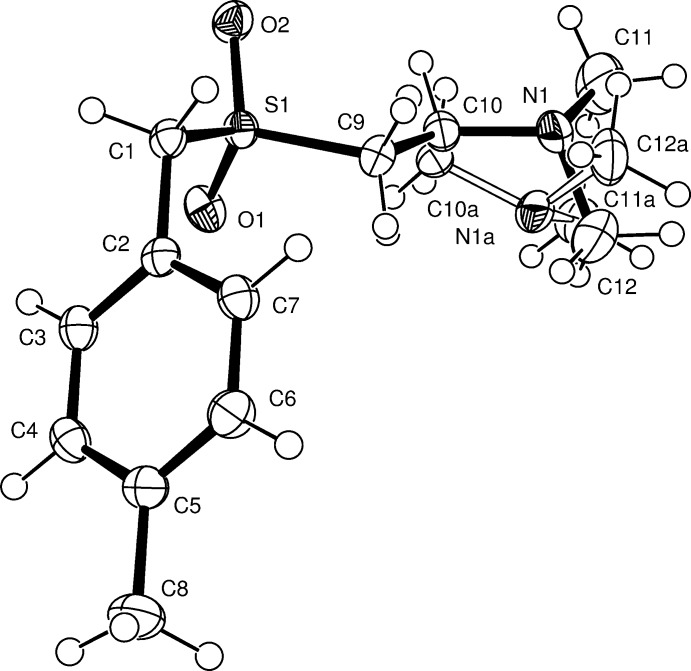
The mol­ecular structure of the title compound with non-H atoms shown as 50% probability displacement ellipsoids. For the disordered fragment, the atoms labelled with the suffix ‘a’ have been shown with hollow bonds whilst all other bonds are shown as solid lines.

The mol­ecular structure of the title compound is shown in Fig. 2 ▸. The amine group is disordered over two equally occupied sites such that the lone pair of the pyramidal N atom is *anti* to O1 with respect to the plane defined by C1—S1—C9 for the conformer containing N1 but *syn* for the N1*A* conformer.

## Supra­molecular features   

**Figure 3:**
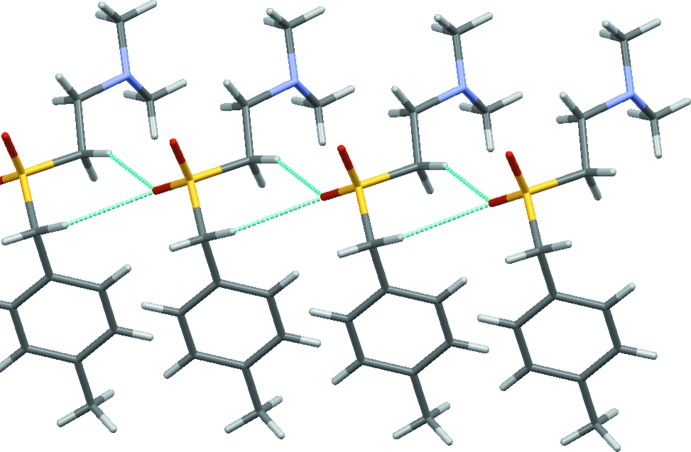
Part of the mol­ecular chain formed by translation along *a* highlighting the C—H⋯O contacts. Only one of the two disordered conformations is shown.

Neighbouring mol­ecules related by translation along the *a*-axis direction are connected by two weak C—H⋯O hydrogen bonds involving O1 and C1 and C9/C9*A* (Table 1 ▸ and Fig. 3 ▸). This gives one-dimensional supra­molecular chains of mol­ecules that propagate parallel to the crystallographic *a*-axis direction.

Other close inter­actions involve the disordered fragment. Thus the methyl group of C11*A* approaches the aromatic ring, giving a C—ċπ interaction [closest contact C6⋯C11*A =* 3.345 (5) Å] whilst C11 forms unfeasibly short inter­molecular inter­actions with its centrosymmetrically related self – an inter­action that is relieved by the observed disorder.

## Synthesis and crystallization   

A mixture of 2-[(4-methyl­benz­yl)sulfon­yl]ethyl methane­sulfonate and 1-methyl-4-[(vinyl­sulfon­yl)meth­yl]benzene (4.880 g) was dissolved in di­chloro­methane (50 ml, dry) to which di­methyl­amine (4 ml, 2*M* in THF) was added at room temperature with stirring. The stirring was continued at room temperature overnight. The reaction mixture was extracted with a saturated solution of sodium carbonate. The organic layer was collected, dried over MgSO_4_, filtered and the solvents were removed under reduced pressure and the crude product was applied to a silica gel column chromatography using first ethyl acetate/*n*-hexane (1/1, RF = 0.1) and then ethyl acetate/methanol (9/1). The product was obtained as a white solid which was recrystallized from ethyl acetate/*n*-hexane (2.200 g) (m.p. 341–343 K). HRESIMS: calculated for C_12_H_19_NO_2_S, 241.1136; found: 241.1139.

## Refinement   

Crystal data, data collection and structure refinement details are summarized in Table 2 ▸. Models where the site occupancy factors of the disordered groups were allowed to refine gave occupancies equal to 50%. So in the final model, occupancies of all the disordered atoms were set to this value. The C9—C10 and C9*A*—C10*A* distances were restrained to be 1.53 (1) Å. All H atoms were placed in idealized positions and were refined in riding modes with C—H equal to 0.95, 0.98 and 0.99 Å for CH, CH_2_ and CH_3_ groups, respectively, and *U*_iso_(H) = 1.5*U*_eq_(C) for methyl groups and 1.2*U*_eq_(C) for all other groups.

## Supplementary Material

Crystal structure: contains datablock(s) I, global. DOI: 10.1107/S2056989015010233/is5399sup1.cif

Structure factors: contains datablock(s) I. DOI: 10.1107/S2056989015010233/is5399Isup2.hkl

Supporting information file. DOI: 10.1107/S2056989015010233/is5399Isup3.cml

CCDC reference: 1403422

Additional supporting information:  crystallographic information; 3D view; checkCIF report

## Figures and Tables

**Table 1:** Hydrogen-bond geometry (, )

*D*H*A*	*D*H	H*A*	*D* *A*	*D*H*A*
C1H1*A*O1^i^	0.99	2.60	3.493(2)	150
C9H9*A*O1^i^	0.99	2.49	3.415(2)	155
C9*A*H9*C*O1^i^	0.99	2.61	3.415(2)	138

Symmetry code: (i) 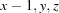.

**Table 2:** Experimental details

Crystal data	
Chemical formula	C_12_H_19_NO_2_S
*M*_r_	241.34
Crystal system, space group	Triclinic, *P* 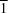
Temperature (K)	123
*a*, *b*, *c* ()	5.3642(3), 10.3773(6), 12.1784(7)
, , ()	99.572(5), 95.498(5), 104.645(5)
*V* (^3^)	639.98(6)
*Z*	2
Radiation type	Cu *K*
(mm^1^)	2.14
Crystal size (mm)	0.30 0.10 0.03

Data collection	
Diffractometer	Oxford Diffraction Gemini S
Absorption correction	Multi-scan (*CrysAlis PRO*; Oxford Diffraction, 2009 ▸)
*T*_min_, *T*_max_	0.459, 0.938
No. of measured, independent and observed [*I* > 2(*I*)] reflections	5846, 2491, 2360
*R*_int_	0.023
(sin /)_max_ (^1^)	0.620

Refinement	
*R*[*F* ^2^ > 2(*F* ^2^)], *wR*(*F* ^2^), *S*	0.043, 0.121, 1.08
No. of reflections	2491
No. of parameters	186
No. of restraints	2
H-atom treatment	H-atom parameters constrained
_max_, _min_ (e ^3^)	0.48, 0.36

Computer programs: *CrysAlis PRO* (Oxford Diffraction, 2009 ▸), *SIR92* (Altomare *et al.*, 1994 ▸), *SHELXL97* (Sheldrick, 2008 ▸), *ORTEP-3 for Windows* (Farrugia, 2012 ▸) and *Mercury* (Macrae *et al.*, 2008 ▸).

## supplementary crystallographic information

### Crystal data

**Table:**

C_12_H_19_NO_2_S	*Z* = 2
*M**_r_* = 241.34	*F*(000) = 260
Triclinic, *P*1	*D*_x_ = 1.252 Mg m^−^^3^
Hall symbol: -P 1	Cu *K*α radiation, λ = 1.54180 Å
*a* = 5.3642 (3) Å	Cell parameters from 3570 reflections
*b* = 10.3773 (6) Å	θ = 5.2–72.9°
*c* = 12.1784 (7) Å	µ = 2.14 mm^−^^1^
α = 99.572 (5)°	*T* = 123 K
β = 95.498 (5)°	Plate, colourless
γ = 104.645 (5)°	0.30 × 0.10 × 0.03 mm
*V* = 639.98 (6) Å^3^	

### Data collection

**Table:**

Oxford Diffraction Gemini S diffractometer	2491 independent reflections
Radiation source: fine-focus sealed tube	2360 reflections with *I* > 2σ(*I*)
Graphite monochromator	*R*_int_ = 0.023
ω scans	θ_max_ = 72.9°, θ_min_ = 3.7°
Absorption correction: multi-scan (*CrysAlis PRO*; Oxford Diffraction, 2009)	*h* = −4→6
*T*_min_ = 0.459, *T*_max_ = 0.938	*k* = −12→11
5846 measured reflections	*l* = −15→14

### Refinement

**Table:**

Refinement on *F*^2^	Primary atom site location: structure-invariant direct methods
Least-squares matrix: full	Secondary atom site location: difference Fourier map
*R*[*F*^2^ > 2σ(*F*^2^)] = 0.043	Hydrogen site location: inferred from neighbouring sites
*wR*(*F*^2^) = 0.121	H-atom parameters constrained
*S* = 1.08	*w* = 1/[σ^2^(*F*_o_^2^) + (0.0758*P*)^2^ + 0.2621*P*] where *P* = (*F*_o_^2^ + 2*F*_c_^2^)/3
2491 reflections	(Δ/σ)_max_ < 0.001
186 parameters	Δρ_max_ = 0.48 e Å^−^^3^
2 restraints	Δρ_min_ = −0.35 e Å^−^^3^

### Special details

**Table:**

Experimental. ^1^H NMR (DMSO-d_6_): δ 7.28 (2*H*, d, J = 8.0 Hz), 7.21 (2*H*, d, J = 8.0 Hz), 4.44 (2*H*, s), 3.17 (2*H*, t, J = 14.3 Hz), 2.65 (2*H*, t, J = 14.3 Hz), 2.31 (3*H*, s), 2.16 (6*H*, s). ^13^C NMR (DMSO-d_6_): δ 137.7, 130.8, 129.0, 125.4, 58.4, 51.6, 49.0, 44.9, 20.7. IR (KBr): 1511, 1463, 1399, 1380, 1314, 1258, 1156, 1119, 1050, 892, 853, 822, 749 cm^-1^.
Geometry. All s.u.'s (except the s.u. in the dihedral angle between two l.s. planes) are estimated using the full covariance matrix. The cell s.u.'s are taken into account individually in the estimation of s.u.'s in distances, angles and torsion angles; correlations between s.u.'s in cell parameters are only used when they are defined by crystal symmetry. An approximate (isotropic) treatment of cell s.u.'s is used for estimating s.u.'s involving l.s. planes.
Refinement. Refinement of *F*^2^ against ALL reflections. The weighted *R*-factor *wR* and goodness of fit *S* are based on *F*^2^, conventional *R*-factors *R* are based on *F*, with *F* set to zero for negative *F*^2^. The threshold expression of *F*^2^ > 2σ(*F*^2^) is used only for calculating *R*-factors(gt) *etc*. and is not relevant to the choice of reflections for refinement. *R*-factors based on *F*^2^ are statistically about twice as large as those based on *F*, and *R*- factors based on ALL data will be even larger.

### Fractional atomic coordinates and isotropic or equivalent isotropic displacement parameters (Å^2^)

**Table:**

	*x*	*y*	*z*	*U*_iso_*/*U*_eq_	Occ. (<1)
S1	0.89090 (7)	0.52015 (4)	0.66118 (3)	0.02154 (16)	
O1	1.1289 (2)	0.48170 (12)	0.68167 (11)	0.0307 (3)	
O2	0.9042 (2)	0.63679 (12)	0.60940 (10)	0.0295 (3)	
C1	0.6462 (3)	0.38079 (16)	0.57471 (13)	0.0223 (3)	
H1A	0.4763	0.4021	0.5740	0.027*	
H1B	0.6866	0.3706	0.4966	0.027*	
C2	0.6217 (3)	0.24813 (16)	0.61229 (13)	0.0211 (3)	
C3	0.7832 (3)	0.16706 (17)	0.57982 (14)	0.0252 (4)	
H3	0.9114	0.1961	0.5338	0.030*	
C4	0.7579 (3)	0.04422 (17)	0.61421 (14)	0.0259 (4)	
H4	0.8692	−0.0101	0.5912	0.031*	
C5	0.5729 (3)	−0.00109 (17)	0.68174 (14)	0.0262 (4)	
C6	0.4119 (4)	0.08042 (18)	0.71320 (16)	0.0300 (4)	
H6	0.2838	0.0513	0.7592	0.036*	
C7	0.4342 (3)	0.20319 (17)	0.67902 (14)	0.0253 (4)	
H7	0.3211	0.2568	0.7012	0.030*	
C8	0.5437 (4)	−0.13542 (19)	0.71811 (18)	0.0377 (5)	
H8A	0.5787	−0.1201	0.8004	0.057*	
H8B	0.6676	−0.1802	0.6856	0.057*	
H8C	0.3659	−0.1933	0.6920	0.057*	
C9	0.7696 (3)	0.55043 (17)	0.79112 (14)	0.0248 (4)	0.50
H9A	0.5883	0.5562	0.7768	0.030*	0.50
H9B	0.7692	0.4741	0.8304	0.030*	0.50
N1	0.8311 (7)	0.7301 (3)	0.9652 (3)	0.0333 (7)	0.50
C10	0.9410 (19)	0.6837 (8)	0.8655 (9)	0.0264 (18)	0.50
H10A	1.1132	0.6711	0.8900	0.032*	0.50
H10B	0.9689	0.7554	0.8200	0.032*	0.50
C11	0.9697 (14)	0.8715 (5)	1.0135 (4)	0.0648 (15)	0.50
H11A	0.8822	0.9060	1.0743	0.097*	0.50
H11B	0.9707	0.9261	0.9551	0.097*	0.50
H11C	1.1492	0.8774	1.0435	0.097*	0.50
C12	0.8399 (16)	0.6456 (6)	1.0475 (5)	0.0683 (18)	0.50
H12A	0.7464	0.5512	1.0134	0.102*	0.50
H12B	0.7577	0.6770	1.1114	0.102*	0.50
H12C	1.0216	0.6513	1.0737	0.102*	0.50
N1A	0.9175 (6)	0.6553 (3)	0.9889 (3)	0.0297 (7)	0.50
C9A	0.7696 (3)	0.55043 (17)	0.79112 (14)	0.0248 (4)	0.50
H9C	0.6106	0.5813	0.7797	0.030*	0.50
H9D	0.7240	0.4654	0.8207	0.030*	0.50
C10A	0.9780 (18)	0.6589 (8)	0.8749 (8)	0.0231 (17)	0.50
H10C	1.1504	0.6423	0.8688	0.028*	0.50
H10D	0.9848	0.7496	0.8578	0.028*	0.50
C11A	1.1294 (9)	0.7511 (4)	1.0688 (3)	0.0394 (9)	0.50
H11D	1.2931	0.7284	1.0582	0.059*	0.50
H11E	1.0940	0.7461	1.1457	0.059*	0.50
H11F	1.1434	0.8434	1.0563	0.059*	0.50
C12A	0.6727 (9)	0.6851 (5)	1.0055 (4)	0.0424 (10)	0.50
H12D	0.6451	0.6851	1.0839	0.064*	0.50
H12E	0.5301	0.6158	0.9554	0.064*	0.50
H12F	0.6772	0.7746	0.9884	0.064*	0.50

### Atomic displacement parameters (Å^2^)

**Table:**

	*U*^11^	*U*^22^	*U*^33^	*U*^12^	*U*^13^	*U*^23^
S1	0.0207 (2)	0.0219 (2)	0.0214 (2)	0.00508 (16)	0.00401 (15)	0.00319 (16)
O1	0.0228 (6)	0.0307 (7)	0.0357 (7)	0.0074 (5)	0.0033 (5)	−0.0006 (5)
O2	0.0357 (7)	0.0258 (6)	0.0264 (6)	0.0050 (5)	0.0073 (5)	0.0071 (5)
C1	0.0221 (8)	0.0237 (8)	0.0190 (7)	0.0053 (6)	0.0001 (6)	0.0018 (6)
C2	0.0199 (7)	0.0219 (8)	0.0182 (7)	0.0031 (6)	−0.0027 (6)	0.0016 (6)
C3	0.0220 (8)	0.0285 (9)	0.0240 (8)	0.0066 (7)	0.0043 (6)	0.0018 (7)
C4	0.0231 (8)	0.0255 (8)	0.0267 (8)	0.0084 (6)	−0.0005 (6)	−0.0011 (7)
C5	0.0261 (8)	0.0231 (8)	0.0258 (8)	0.0039 (6)	−0.0023 (6)	0.0031 (6)
C6	0.0275 (9)	0.0309 (9)	0.0327 (9)	0.0060 (7)	0.0102 (7)	0.0094 (7)
C7	0.0218 (8)	0.0274 (8)	0.0265 (8)	0.0079 (7)	0.0041 (6)	0.0029 (7)
C8	0.0437 (11)	0.0273 (9)	0.0433 (11)	0.0098 (8)	0.0056 (9)	0.0110 (8)
C9	0.0252 (8)	0.0284 (8)	0.0200 (8)	0.0062 (7)	0.0042 (6)	0.0043 (6)
N1	0.045 (2)	0.0337 (17)	0.0244 (16)	0.0202 (16)	0.0042 (15)	0.0002 (13)
C10	0.024 (3)	0.034 (3)	0.022 (3)	0.013 (2)	−0.0005 (19)	0.001 (2)
C11	0.106 (5)	0.042 (3)	0.037 (2)	0.017 (3)	0.006 (3)	−0.011 (2)
C12	0.121 (6)	0.066 (4)	0.033 (3)	0.045 (4)	0.025 (3)	0.016 (3)
N1A	0.0366 (17)	0.0302 (17)	0.0197 (17)	0.0083 (14)	−0.0003 (13)	0.0013 (13)
C9A	0.0252 (8)	0.0284 (8)	0.0200 (8)	0.0062 (7)	0.0042 (6)	0.0043 (6)
C10A	0.023 (3)	0.027 (3)	0.019 (2)	0.009 (2)	−0.0031 (18)	0.003 (2)
C11A	0.046 (2)	0.040 (2)	0.0253 (18)	0.0098 (18)	−0.0103 (16)	−0.0028 (16)
C12A	0.046 (2)	0.053 (3)	0.028 (2)	0.020 (2)	0.0083 (19)	−0.0024 (18)

### Geometric parameters (Å, º)

**Table:**

S1—O1	1.4426 (13)	C9—H9B	0.9900
S1—O2	1.4446 (12)	N1—C12	1.442 (6)
S1—C9	1.7780 (16)	N1—C11	1.460 (6)
S1—C1	1.7867 (16)	N1—C10	1.462 (12)
C1—C2	1.501 (2)	C10—H10A	0.9900
C1—H1A	0.9900	C10—H10B	0.9900
C1—H1B	0.9900	C11—H11A	0.9800
C2—C7	1.391 (2)	C11—H11B	0.9800
C2—C3	1.392 (2)	C11—H11C	0.9800
C3—C4	1.386 (2)	C12—H12A	0.9800
C3—H3	0.9500	C12—H12B	0.9800
C4—C5	1.390 (3)	C12—H12C	0.9800
C4—H4	0.9500	N1A—C12A	1.448 (6)
C5—C6	1.390 (2)	N1A—C11A	1.457 (5)
C5—C8	1.507 (2)	N1A—C10A	1.460 (12)
C6—C7	1.385 (2)	C10A—H10C	0.9900
C6—H6	0.9500	C10A—H10D	0.9900
C7—H7	0.9500	C11A—H11D	0.9800
C8—H8A	0.9800	C11A—H11E	0.9800
C8—H8B	0.9800	C11A—H11F	0.9800
C8—H8C	0.9800	C12A—H12D	0.9800
C9—C10	1.536 (7)	C12A—H12E	0.9800
C9—H9A	0.9900	C12A—H12F	0.9800
			
O1—S1—O2	117.10 (8)	H8B—C8—H8C	109.5
O1—S1—C9	108.51 (8)	C10—C9—S1	109.9 (5)
O2—S1—C9	108.29 (8)	C10—C9—H9A	109.7
O1—S1—C1	109.89 (7)	S1—C9—H9A	109.7
O2—S1—C1	107.22 (7)	C10—C9—H9B	109.7
C9—S1—C1	105.17 (8)	S1—C9—H9B	109.7
C2—C1—S1	113.98 (11)	H9A—C9—H9B	108.2
C2—C1—H1A	108.8	C12—N1—C11	110.8 (4)
S1—C1—H1A	108.8	C12—N1—C10	111.7 (5)
C2—C1—H1B	108.8	C11—N1—C10	109.5 (5)
S1—C1—H1B	108.8	N1—C10—C9	113.9 (8)
H1A—C1—H1B	107.7	N1—C10—H10A	108.8
C7—C2—C3	118.87 (15)	C9—C10—H10A	108.8
C7—C2—C1	120.38 (14)	N1—C10—H10B	108.8
C3—C2—C1	120.74 (14)	C9—C10—H10B	108.8
C4—C3—C2	120.35 (15)	H10A—C10—H10B	107.7
C4—C3—H3	119.8	C12A—N1A—C11A	110.2 (3)
C2—C3—H3	119.8	C12A—N1A—C10A	112.8 (4)
C3—C4—C5	121.28 (15)	C11A—N1A—C10A	109.0 (4)
C3—C4—H4	119.4	N1A—C10A—H10C	109.8
C5—C4—H4	119.4	N1A—C10A—H10D	109.8
C4—C5—C6	117.86 (16)	H10C—C10A—H10D	108.2
C4—C5—C8	121.24 (16)	N1A—C11A—H11D	109.5
C6—C5—C8	120.90 (16)	N1A—C11A—H11E	109.5
C7—C6—C5	121.50 (16)	H11D—C11A—H11E	109.5
C7—C6—H6	119.3	N1A—C11A—H11F	109.5
C5—C6—H6	119.3	H11D—C11A—H11F	109.5
C6—C7—C2	120.15 (15)	H11E—C11A—H11F	109.5
C6—C7—H7	119.9	N1A—C12A—H12D	109.5
C2—C7—H7	119.9	N1A—C12A—H12E	109.5
C5—C8—H8A	109.5	H12D—C12A—H12E	109.5
C5—C8—H8B	109.5	N1A—C12A—H12F	109.5
H8A—C8—H8B	109.5	H12D—C12A—H12F	109.5
C5—C8—H8C	109.5	H12E—C12A—H12F	109.5
H8A—C8—H8C	109.5		
			
O1—S1—C1—C2	47.30 (14)	C8—C5—C6—C7	−179.06 (16)
O2—S1—C1—C2	175.57 (11)	C5—C6—C7—C2	−0.4 (3)
C9—S1—C1—C2	−69.31 (13)	C3—C2—C7—C6	0.7 (2)
S1—C1—C2—C7	96.45 (16)	C1—C2—C7—C6	179.81 (14)
S1—C1—C2—C3	−84.47 (17)	O1—S1—C9—C10	72.2 (3)
C7—C2—C3—C4	−0.4 (2)	O2—S1—C9—C10	−55.9 (3)
C1—C2—C3—C4	−179.51 (14)	C1—S1—C9—C10	−170.2 (3)
C2—C3—C4—C5	−0.2 (3)	C12—N1—C10—C9	71.5 (7)
C3—C4—C5—C6	0.5 (3)	C11—N1—C10—C9	−165.3 (5)
C3—C4—C5—C8	179.36 (16)	S1—C9—C10—N1	169.5 (4)
C4—C5—C6—C7	−0.2 (3)		

### Hydrogen-bond geometry (Å, º)

**Table:**

*D*—H···*A*	*D*—H	H···*A*	*D*···*A*	*D*—H···*A*
C1—H1*A*···O1^i^	0.99	2.60	3.493 (2)	150
C9—H9*A*···O1^i^	0.99	2.49	3.415 (2)	155
C9*A*—H9*C*···O1^i^	0.99	2.61	3.415 (2)	138

Symmetry code: (i) *x*−1, *y*, *z*.
